# Adherence to a procalcitonin-guided antibiotic treatment protocol in patients with severe sepsis and septic shock

**DOI:** 10.1186/s13613-018-0415-5

**Published:** 2018-06-04

**Authors:** Andreas Hohn, Nina Balfer, Bernhard Heising, Sabine Hertel, Jan C. Wiemer, Marcel Hochreiter, Stefan Schröder

**Affiliations:** 1 Department of Anaesthesiology and Intensive Care Medicine, University Hospital of Cologne, Kerpener Str. 62, 50937 Cologne, Germany; 2 Department of Infectiology and Hospital Hygiene, Hospital Düren gem. GmbH, Roonstraße 30, 52351 Düren, Germany; 3 Thermo Fisher Scientific, Thermo Scientific Biomarkers, Neuendorfstr. 25, 16761 Hennigsdorf, Germany; 4 Department of Anaesthesiology, University Hospital Heidelberg, Im Neuenheimer Feld 110, 69120 Heidelberg, Germany; 5 Department of Anaesthesiology, Surgical Intensive Care, Emergency Medicine and Pain Management, Hospital Düren gem. GmbH, Roonstraße 30, 52351 Düren, Germany

**Keywords:** Sepsis, Procalcitonin, Antibiotic consumption, Protocol adherence

## Abstract

**Background:**

In randomised controlled trials, procalcitonin (PCT)-guided antibiotic treatment has been proven to significantly reduce length of antibiotic therapy in intensive care unit (ICU) patients. However, concern was raised on low protocol adherence and high rates of overruling, and thus the value of PCT-guided treatment in real clinical life outside study conditions remains unclear. In this study, adherence to a PCT protocol to guide antibiotic treatment in patients with severe sepsis and septic shock was analysed.

**Methods:**

From 2012 to 2014, surgical ICU patients with severe sepsis or septic shock were retrospectively screened for PCT measurement series appropriate to make treatment decisions on antibiotic therapy. We compared (1) patients with appropriate PCT measurement series to patients without appropriate series; (2) patients who reached the antibiotic stopping advice threshold (PCT < 0.5 ng/mL and/or decrease to 10% of peak level) to patients who did not reach a stopping advice threshold; and (3) patients who were treated adherently to the PCT protocol to non-adherently treated patients. The groups were compared in terms of antibiotic treatment duration, PCT kinetics, and other clinical outcomes.

**Results:**

Of 81 patients with severe sepsis or septic shock, 14 were excluded due to treatment restriction or short course in the ICU. The final analysis was performed on 67 patients. Forty-two patients (62.7%) had appropriate PCT measurement series. In patients with appropriate PCT series, median initial PCT (*p* = 0.001) and peak PCT levels (*p* < 0.001) were significantly higher compared to those with non-appropriate series. In 26 patients with appropriate series, PCT levels reached an antibiotic stopping advice. In 8 of 26 patients with stopping advice, antibiotics were discontinued adherently to the PCT protocol (30.8%). Patients with adherently discontinued antibiotics had a shorter antibiotic treatment (7d [IQR 6–9] vs. 12d [IQR 9–16]; *p* = 0.002). No differences were seen in terms of other clinical outcomes.

**Conclusion:**

In patients with severe sepsis and septic shock, procalcitonin testing was irregular and adherence to a local PCT protocol was low in real clinical life. However, adherently treated patients had a shorter duration of antibiotic treatment without negative clinical outcomes. Procalcitonin peak values and kinetics had a clear impact on the regularity of PCT testing.

**Electronic supplementary material:**

The online version of this article (10.1186/s13613-018-0415-5) contains supplementary material, which is available to authorized users.

## Background

Length of antibiotic therapy in critically ill patients can be safely guided by PCT-guided protocols. A recent large study and several reviews and meta-analyses demonstrated that implementation of PCT-guided protocols leads to a significant reduction in antibiotic exposure of 2–3.5 days [1–8]. Despite this reduction, a recent Cochrane Database review found no beneficial effects on mortality, duration of mechanical ventilation, or reinfection [9]. However, the use of PCT to guide treatment decisions is recommended in current guidelines [10, 11].

Concern was raised on low protocol adherence in prospective trials. The low rate of compliance of PCT-guided algorithms and the high rate of exclusion weaken the real impact of such protocols in the clinical decision-making process [12]. However, own data revealed that in clinical routine, duration of antibiotic therapy in septic ICU patients decreased after implementation of a PCT protocol [13], and combination with an antibiotic stewardship programme had positive impact on antibiotic use density and the spectrum of antibiotic classes used in the ICU [14]. Nonetheless, data are scarce regarding adherence to PCT protocols for guidance of antibiotic treatment. A retrospective analysis from seven German intensive care units (ICU) showed that the use of PCT measurement to monitor sepsis treatment is not well established in clinical routine [15]. The aim of this retrospective cohort study from a surgical ICU was to analyse adherence to a PCT-guided antibiotic treatment protocol in patients with severe sepsis and septic shock. Furthermore, we sought to assess factors affecting protocol adherence, to get a better understanding on the use of PCT in real clinical life.

## Methods

In brief, patients with severe sepsis and septic shock were retrospectively identified by a database query from the hospital information system by their primary diagnosis (i.e. severe sepsis/septic shock). In septic patients, procalcitonin measurement series were analysed to assess whether measurement series were appropriate to guide antibiotic treatment or not. Patients with appropriate PCT measurement series were compared to patients without appropriate series in terms of antibiotic treatment duration, PCT kinetics, and other clinical outcomes. Afterwards, among patients with appropriate PCT measurement series, those with PCT stopping advice (PCT < 0.5 ng/mL and/or decrease to 10% of peak level) to discontinue antibiotics according to the local PCT protocol were compared to patients which did not reach a stop threshold.

If antibiotics were discontinued when a PCT stopping advice was reached, patients were classified as being treated adherently to the PCT protocol. Finally, adherently treated patients were compared to non-adherently treated patients again in terms of duration of antibiotic treatment, PCT kinetics, and other clinical outcomes.

### Study design and patients

In this retrospective cohort study, patients admitted to the surgical intensive care unit (ICU) between 2012 and 2014 with the diagnosis of severe sepsis or septic shock were included. To define the first episode of sepsis, the time of sepsis diagnosis and the source of infection, data were taken from a review of patient records by four of the authors. Furthermore, data on antibiotic treatment were analysed in the same way. Information on the following variables was available from the hospital information system and the patients’ chart:

- Age, gender and Simplified Acute Physiology Score (SAPS) II at admission to ICU.
- Maximum sequential organ failure score (SOFA).
- Type of infection.
- Length of stay in ICU and length of stay in hospital.
- PCT measurements and results during ICU stay.
- Duration of mechanical ventilation.
- Doses of antibiotics and antibiotic use density during ICU stay.
- ICU survival status.
- Final diagnosis at discharge.

### Description of the local PCT protocol and PCT stopping advice

Procalcitonin-guided antibiotic treatment was introduced in our ICU in 2011 [14]. The PCT protocol used from 2012 contains the following rules:

- Initiation of antibiotic therapy based on clinical decision.
- Daily PCT (day 1–3) measurement in patients undergoing antibiotic treatment started on admission to ICU or clinical diagnosis of sepsis, severe sepsis or septic shock.
- From day 4 on, PCT samples every other day in patients under antibiotic treatment.

Antibiotic stopping advice: If PCT is < 0.50 ng/mL or PCT decreases to ≤ 10% from peak level, discontinuation of antibiotic treatment is recommended. If PCT is ≥ 0.5 ng/mL and does not decrease to ≤ 10% of peak level, or even increases, discontinuation of antibiotics is only suggested assuming other conditions (unrelated to bacterial infections) leading to increased PCT levels (Additional file 1: Figure S1).

### Definition of appropriate PCT measurement series

The local PCT protocol recommended daily PCT measurements in patients under antibiotic treatment on day 1–3, and PCT measurements every other day from day 4 on. However, since it is not mandatory to have daily PCT measurements, to guide antibiotic treatment sufficiently, we decided that it was appropriate if PCT results were available at regular intervals (e.g. on day 2, 3, or 4, and then on 5, 6, 7, or 8 etc.).

**Fig. 1:**
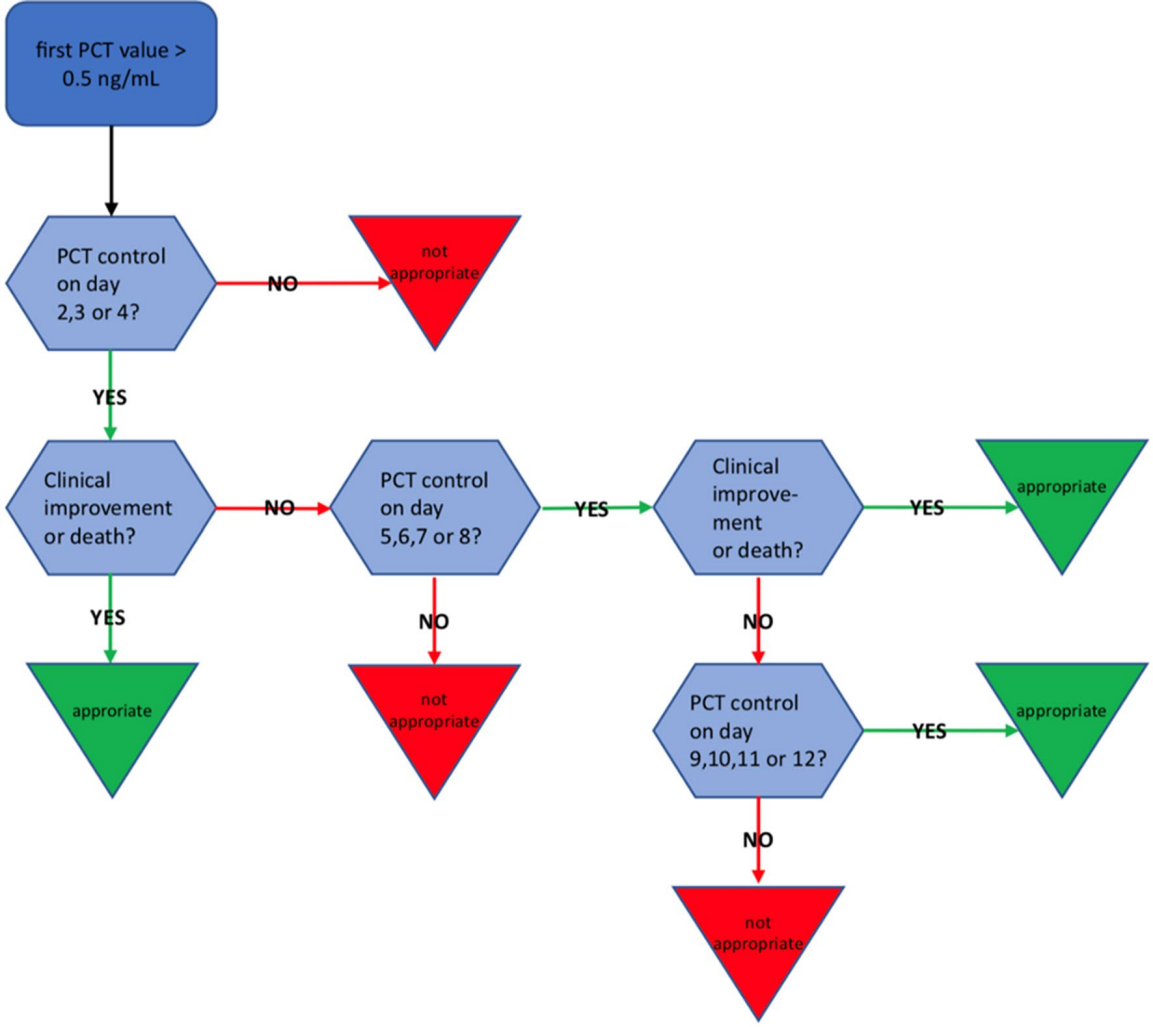
Selection algorithm to identify patients with appropriate procalcitonin (PCT) measurement series according to [15]

A selection of patients with appropriate PCT measurement series was then done accordingly to a recently published analysis [15] (Fig. 1). Patients with restriction of critical care treatment, or short stay (≤ 1–2 days) in the ICU, were removed from the analysis beforehand. Antibiotic therapy and PCT measurements were evaluated only for the first antibiotic episode of infection.

### Definition of adherence to the PCT protocol

According to the implemented PCT algorithm, patients with appropriate PCT measurement series were considered to have been treated adherently if antibiotics were discontinued within 24 h after a PCT decrease to ≤ 10% from peak level or a PCT value declining to < 0.5 ng/mL. The PCT decrease was calculated by the following formula:

$${\text{PCT}}\;{\text{decrease}}\;{\text{at}}\;{\text{day}}\;i\; = \;\frac{{\hbox{max} \left( {{\text{PCT}}\;{\text{before}}\;{\text{day}}\;i} \right) - {\text{PCT}}\;{\text{at}}\;{\text{day}}\;i }}{{\hbox{max} \left( {{\text{PCT}}\;{\text{before}}\;{\text{day}}\;i} \right)}}$$

The PCT value at day *i* was compared to all available PCT values at the days before.

### Group comparisons and statistical analysis

Comparisons regarding antibiotic treatment duration, PCT kinetics, and other clinical outcomes were performed for three different groups of patients:

1. Patients with appropriate PCT measurement series versus patients without appropriate PCT measurement series.
2. Patients who reached antibiotic stopping advice versus patients who did not reach antibiotic stopping advice according to the local PCT protocol.
3. For patients who reached antibiotic stopping advice: Patients treated adherently to the PCT protocol versus patients treated non-adherently (i.e. antibiotics stopped vs. not stopped when PCT stopping advice occurred).

Variables were analysed by

- Counts and proportions for categorical variables,
- means and standard deviations and medians and quartiles for numerical variables.

95% confidence intervals for proportions were determined with the exact method of Clopper and Pearson. Pearson’s *χ*^2^ test was applied for group comparisons of binary variables. The Wilcoxon rank-sum test was applied for comparing two groups, and p values are reported. Differences with *p* < 0.05 were considered statistically significant. All statistical analyses were performed with *R* version 3.1.2 (*R* Foundation for Statistical Computing, Vienna, Austria).

## Results

**Fig. 2:**
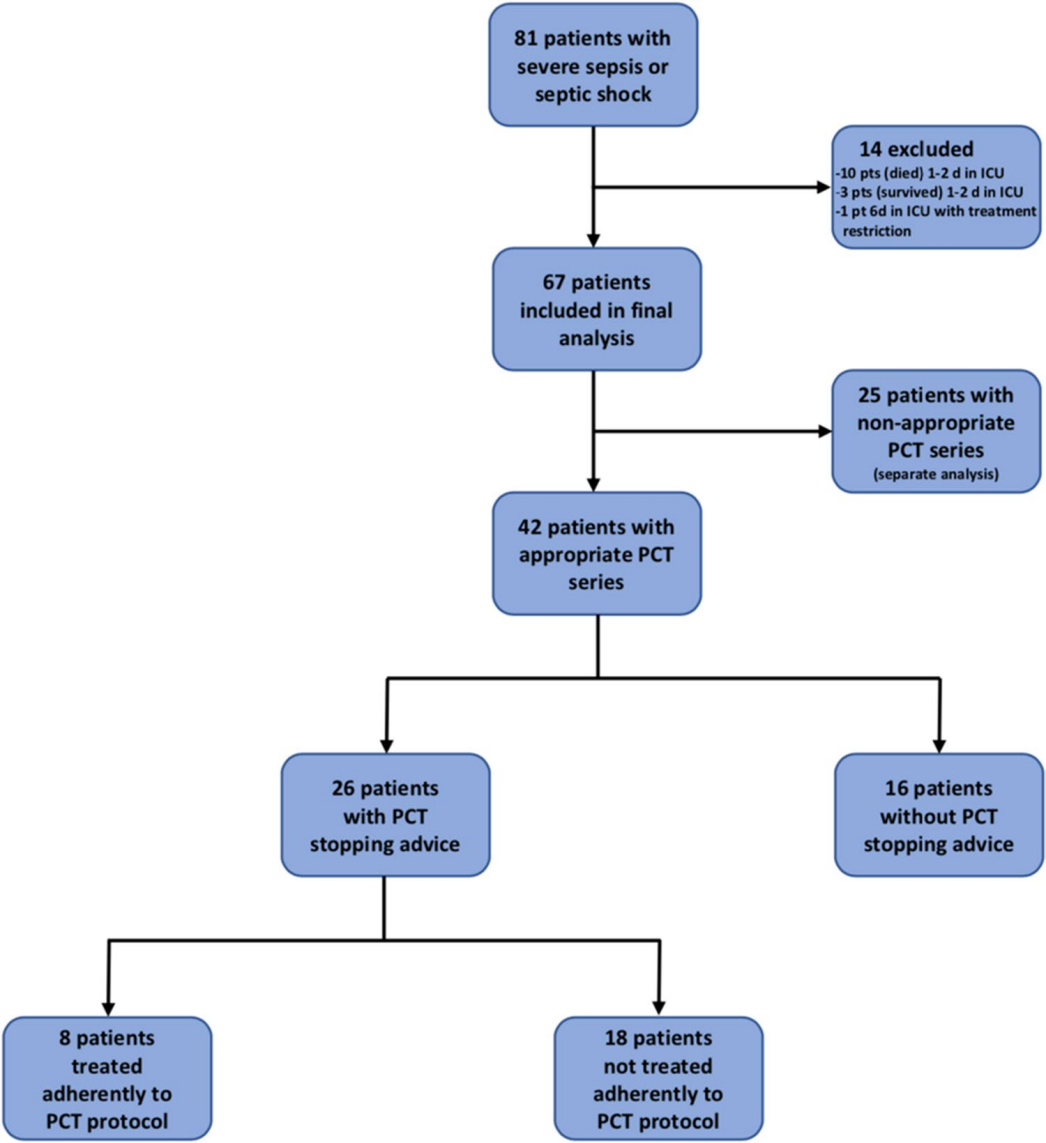
Flow diagram of septic ICU patients and distribution to different subgroups of procalcitonin testing and protocol adherence. *pt(s)* patient(s)

Between 2012 and 2014, 81 patients with the final diagnosis of severe sepsis and septic shock were identified from the hospital information system. Of these, 14 were excluded due to treatment restriction or short stay in the ICU (Fig. 2). The final analysis was performed for 67 patients. According to our definition (Fig. 1), 25 patients (37.3%) did not have appropriate PCT measurement series. Of the remaining 42 patients, 26 patients reached an antibiotic stopping advice threshold according to the local PCT protocol. In 8 cases (30.8%) antibiotics were stopped adherently to the PCT protocol.

### Factors affecting appropriate versus non-appropriate PCT measurement series

Table 1 shows the patient characteristics and outcomes of patients with appropriate PCT measurement series (*n* = 42) compared to those with non-appropriate PCT measurement series (*n* = 25). Patients with appropriate PCT measurement series had statistically significant longer ventilation times and a longer duration of antibiotic treatment in the first episode of sepsis. ICU mortality was lower in the group with non-appropriate PCT series, and the rate of pneumonias was higher in the non-appropriate group.

**Table 1:** Demographics and clinical data of patients with appropriate and non-appropriate procalcitonin (PCT) measurement series

	Patients with appropriate PCT series (*n* = 42)	Patients with non-appropriate PCT series (*n* = 25)	*p* value
Age, years (median [IQR])	68 [59–76]	76 [68–79]	0.172
SAPS II score (median [IQR])	40 [29–51]	38 [32–47]	0.807
Male gender (% [95% CI])	59.5 [43.3–74.4]	60.0 [38.7–78.9]	0.999
ICU mortality (% [95% CI])	40.5 [26–57]	32.0 [14.9–53.5]	0.604
Ventilation hours (median [IQR])	423 [68–840]	102 [8–335]	0.012
ICU LOS, days (median [IQR])	17 [5–41]	11 [4–21]	0.264
Hospital LOS, days (median [IQR])	42 [25–73]	39 [25–54]	0.351
SOFA score max (median [IQR])	11 [9–13]	9 [8–11]	0.112
Length of antibiotic treatment, days (median [IQR])	10 [7–13]	5 [4–6]	< 0.001
Antibiotic use density, DDD (median [IQR])	11 [9–23]	6 [4–9]	< 0.001
Initial PCT value, ng/mL (median [IQR])	9.15 [3.1–32.4]	1.55 [0.9–4.6]	0.001
Peak PCT value, ng/mL (median [IQR])	30.2 [7.1–60.7]	3.8 [1.6–10.3]	< 0.001

	% [95% CI] (*n*)	% [95% CI] (*n*)
*Cause of infection*		
Abdominal sepsis	52.4 [36.4–67.9] (22)	44.0 [24.4–65.1] (11)
Bloodstream infection	0	4.0 [0.1–20.3] (1)
Pleural empyema	9.5 [2.6–22.6] (4)	0
Pneumonia	19.0 [8.6–34.1] (8)	32.0 [14.9–53.5] (8)
Prosthetic infection	0	8.0 [1.0–26.0] (2)
Urogenital sepsis	9.5 [2.6–22.6] (4)	8.0 [1.0–26.0] (2)
Soft tissue infection	2.4 [0.6–12.6] (1)	4.0 [0.1–20.3] (1)
Unknown	7.1 [1–19] (3)	0

Median initial and peak PCT values of the first septic episode were statistically significant higher in the group of patients with appropriate PCT measurement series.

### Factors affecting achievement of PCT stopping advice threshold

Table 2 gives the patient characteristics and outcomes of patients who reached an antibiotic stopping advice threshold according to the PCT protocol (*n* = 26) compared to those without reaching the stopping advice threshold (*n* = 16).

**Table 2:** Demographics and clinical data of patients with and without procalcitonin (PCT) stopping advice

	Patients with PCT stopping advice (*n* = 26)	Patients without PCT stopping advice (*n* = 16)	*p* value
Age, years (median [IQR])	68 [59–75]	70 [62–79]	0.475
SAPS II score (median [IQR])	40 [29–51]	44 [33–52]	0.597
Male gender (% [95% CI])	69.2 [48.2–85.7]	43.8 [19.8–70.1]	0.121
ICU mortality (% [95% CI])	26.9 [11.6–47.8]	62.5 [35.4–84.8]	0.029
Ventilation hours (median [IQR])	597 [96–1260]	238 [47–461]	0.068
ICU LOS, days (median [IQR])	23 [5–56]	11 [5–20]	0.212
Hospital LOS, days (median [IQR])	56 [35–100]	23 [17–44]	0.002
SOFA score max (median [IQR])	11 [9–12]	13 [9–15]	0.320
Length of antibiotic treatment, days (median [IQR])	10 [8–13]	10 [7–12]	0.398
Antibiotic use density, DDD (median [IQR])	13 [10–24]	10 [8–15]	0.136
Initial PCT value, ng/mL (median [IQR])	20.0 [3.3–42.7]	5.1 [2.8–10.8]	0.136
Peak PCT value, ng/mL (median [IQR])	40.5 [9.2–69.7]	16.2 [6.0–32.5]	0.095

	% [95% CI] (*n*)	% [95% CI] (*n*)
*Cause of infection*		
Abdominal sepsis	57.7 [36.9,76.6] (15)	43.8 [19.8–70.1] (7)
Bloodstream infection	0	0
Pleural empyema	11.5 [2.4–30.2] (3)	6.3 [0.2–30.2] (1)
Pneumonia	26.9 [11.5–47.8] (7)	6.3 [0.2–30.2] (1)
Prosthetic infection	0	0
Urogenital sepsis	3.8 [0.1–19.6] (1)	18.8 [4.0–45.6] (3)
Soft tissue infection	0	6.3 [0.2–30.2] (1)
Unknown	0	18.8 [4.0–45.6] (3)

Patients who reached the antibiotic stopping advice had a statistically significant longer stay in the hospital and a relevant lower ICU mortality. SAPS II and maximum SOFA scores were higher in patients who did not reach the stopping advice. Median initial and peak PCT values were higher in patients with stopping advice, but these differences were not statistically significant. Length of antibiotic therapy was comparable in both groups. In 24 of 26 cases, patients reached the stopping advice by a 90% decrease in PCT. A 90% decrease plus PCT < 0.5 ng/mL occurred only in 2 of 26 patients. A drop of PCT below < 0.5 ng/mL alone was in none of the cases a reason for a stopping advice. In patients who did not reach a stopping advice in the PCT protocol, a secondary PCT increase occurred in 81.3% and only in 42.3% of patients who reached the stopping advice threshold.

### Factors affecting adherence to the PCT protocol

Table 3 shows the patient characteristics and outcomes of patients treated adherently to the antibiotic stopping advice (*n* = 8) compared to those with non-adherent treatment (*n* = 18).

**Table 3:** Demographics and clinical data of patients with procalcitonin (PCT) stopping advice treated adherently and non-adherently to the PCT protocol

	Patients adherent to PCT stopping advice (*n* = 8)	Patients non-adherent to PCT stopping advice (*n* = 18)	*p* value
Age, years (median [IQR])	67 [59–75]	68 [59–75]	0.560
SAPS II score (median [IQR])	35 [31–43]	44 [28–51]	0.655
Male gender (% [95% CI])	50.0 [15.7–84.3]	77.8 [30.6–69.4]	0.197
ICU mortality (% [95% CI])	12.5 [0.3–52.7]	33.3 [13.3–59.0]	0.375
Ventilation hours (median [IQR])	353 [68–621]	735 [209–1508]	0.317
ICU LOS, days (median [IQR])	24 [3–38]	23 [12–59]	0.780
Hospital LOS, days (median [IQR])	57 [47–79]	54 [34–100]	0.824
SOFA score max (median [IQR])	11 [9–12]	12 [9–13]	0.557
Length of antibiotic treatment, days (median [IQR])	7 [6–9]	12 [9–16]	0.002
Antibiotic use density, DDD (median [IQR])	10 [7–12]	18 [12–26]	0.001
Initial PCT value, ng/mL (median [IQR])	20.0 [9.4–38.2]	16.8 [2.0–42.7]	0.560
Peak PCT value, ng/mL (median [IQR])	32.3 [9.4–64.2]	44.9 [13.9–69.7]	0.718

	% [95% CI] (*n*)	% [95% CI] (*n*)
*Cause of infection*		
Abdominal sepsis	87.5 [47.3–99.7] (7)	44.4 [21.5–69.2] (8)
Bloodstream infection	0	0
Pleural empyema	0	16.7 [3.5–41.4] (3)
Pneumonia	12.5 [0.3–52.7] (1)	33.3 [13.3–59.0] (6)
Prosthetic infection	0	0
Urogenital sepsis	0	5.6 [0.1–27] (1)
Soft tissue infection	0	0
Unknown	0	0

Both groups were comparable with respect to SAPS II, maximum SOFA scores, length of hospital and ICU stay, and ventilation hours. The rate of male patients was lower in the adherent group, and median length of antibiotic treatment was statistically significant shorter (7 vs. 12 days).

In the adherently treated group, 88% per cent had abdominal sepsis. A trend was seen towards a lower ICU mortality and higher median initial PCT values in adherently treated patients. In 75% of adherently treated patients, the initial PCT value and peak value were concordant, whereas in non-adherent patients only in 44% the initial and peak values of PCT were concordant.

## Discussion

### Factors affecting adherence to the PCT protocol

Currently, there are only limited data on PCT protocol adherence in critically ill patients outside of study conditions. One study in medical ICU patients revealed an adherence below 50% to a local PCT protocol [16]. However, this study did not compare adherently treated patients to non-adherently treated patients. Thus, our study reveals important insights on whether and how the promising strategy of PCT-guidance is transferred to clinical routine practice.

We detected a low adherence to discontinue antibiotics adherently to a PCT stopping advice in clinical real life. In only 8 out of 81 patients with severe sepsis and septic shock during the entire study period, or in 67 patients who entered the final statistical analysis, the algorithm was fully applied. However, in patients with a low PCT (< 0.5 ng/mL) or a marked decrease of PCT levels (≤ 10% of peak level), a shorter antibiotic treatment was not associated with worse clinical outcomes. Thus, our study confirms the results of a recent large study [1] and several reviews and meta-analyses [2–6]. Procalcitonin is able to support clinical decision-making to discontinue antibiotics when PCT decrease goes along with clinical improvement. However, in our study, antibiotics were continued in nearly 70% of patients although antibiotic discontinuation was recommended by the PCT protocol. Algorithm overruling was also of concern in prospective trials where overruling rates reached from 16 to 53% [17–19]. Expectably, overruling rates were higher in real life since the PCT protocol was not applied under controlled study conditions and populations are not comparable due to strict in- and exclusion criteria in randomised trials. Exclusion rates in randomised PCT studies range from 0.3% [20] to 84% [21], thus not necessarily reflecting clinical real life. For example, patients with common ICU infections, e.g. caused by *Pseudomonas aeruginosa* and *Acinetobacter baumannii*, which were excluded in several studies [17–19] were not excluded in our study. Furthermore, probably we cannot exclude that inappropriate septic source control contributed to low adherence to antibiotic stopping advices in our study, and we did not analyse the impact of multi drug resistant pathogens on antibiotic treatment duration.

Nonetheless, the low rate of less than 10% of septic patients in which discontinuation of antibiotics was accompanied by a PCT stop signal challenges the clinical relevance of PCT-guided antibiotic stewardship and efforts should be made to improve adherence to PCT protocols in clinical practice.

Kinetics of procalcitonin also affect adherence to discontinue antibiotics when a stopping advice threshold is reached. Median initial PCT levels were higher in adherently treated patients. In 75% of the adherently treated patients, the initial PCT corresponded to the peak value. Contrasting to that, in 56% of the not adherently treated patients the PCT value rises after the initial value. For clinicians, high initial PCT levels seem to be no barrier to stop antibiotics according to the PCT protocol, but secondarily increasing PCT seems to prompt clinicians to continue antibiotics. Similarly, to the study by Jensen [20], in clinical real life, secondary increases seem to be interpreted as “alert PCT”. For most patients, a decline of PCT to ≤ 10% of peak value was a trigger for an antibiotic stopping advice and not the absolute limit of PCT < 0.5 ng/mL. Thus, in patients with severe sepsis and septic shock, relative variations of PCT seem to be more important to the clinician than absolute values. Furthermore, in patients with secondary PCT increases, the procalcitonin protocol was more frequently overruled and antibiotic treatment continued despite reaching a stopping advice.

Our data suggest that certain types of infection like pneumonia or abdominal sepsis might affect adherence to PCT protocols as well. In patients who reached a stopping advice, those with abdominal sepsis were treated adherently in 43% cases (7/15), whereas patients with pneumonia were treated adherently only in 14% (1/7). However, due to the small number of patients with other infection sites than abdomen or lung, this could not be proven statistically in this study. Nonetheless, these results are interesting since mortality in critically ill septic patients with pneumonia or abdominal sepsis are comparable [22, 23].

### Factors affecting appropriate versus non-appropriate PCT measurement series

A prerequisite to PCT-guided antibiotic stewardship is regular measurements. In our study, 62.7% had appropriate PCT measurement series. Median initial PCT values and peak values were statistically significantly higher in the group with appropriate measurement series. These results suggest that frequency of PCT testing is affected by PCT levels, and high PCT values might prompt clinicians to request PCT testing more frequently. In the group of patients with non-appropriate PCT series, there was a higher number of patients with pneumonia. It is well known that peak values of PCT are lower in respiratory tract infections compared to other kind of infections [24–26]. Lower duration of mechanical ventilation, shorter stay in the ICU, and lower mortality rates reflect that patients without appropriate PCT measurement series were in a better clinical condition, and probably regularly PCT testing was not considered necessary by the clinician. In patients with inappropriate PCT series, median duration of antibiotic treatment was 5 days, and thus more regular PCT testing likely would not have had beneficial impact on further reduction in antibiotic exposure.

### Factors affecting achievement of PCT stopping advice threshold

Beside its value for biomarker-guided antibiotic stewardship, PCT can be used as a prognostic value. Patients who did not reach the PCT stopping advice threshold had higher mortality rates (63%) compared to those where PCT declined indicating that the infection is under control (27%). Median initial PCT levels and peak levels were higher in patients with stopping advice. However, these results were statistically not significant. Interestingly, in 81.3% (13/16) of patients without reaching the PCT stopping advice, PCT levels increased after the initial value. In patients with stopping advice, this occurred only in 42.3% (11/26). These results are in line with previous studies which showed that high PCT levels are associated with an unfavourable outcome [27, 28]. Furthermore, a not adequately declining PCT is an independent predictor of mortality in septic patients [29, 30]. In our study, the shorter ICU and hospital lengths of stay in patients without stopping advice might be founded in the fact that those patients had a higher mortality.

### Limitations

There are limitations of the present study which should be mentioned. Taken the overall low sample size together with the retrospective study design, all results and their interpretation should be taken with caution. As possibly not all septic cases might have been registered correctly in the hospital’s database, we cannot exclude a selection bias in our analysis. The selection of patients was also possibly affected by the definition of an appropriate measurement series. However, today, there is no universal definition how often and in what intervals PCT should be measured to be sufficient to guide antibiotic treatment. In addition, there are little data for the frequency of PCT testing and adherence to PCT protocols in clinical real life. In our study, we used a recently published definition to classify a series of PCT measurements as appropriate to guide antibiotic treatment [15]. Furthermore, we can only assess correlation and cannot comment on causality, and thus, probably additional factors may have contributed to the results.

Furthermore, we can only comment on the first episode of sepsis and did not analyse the impact of PCT guidance on further septic episodes during the stay in ICU. As our data were collected retrospectively, diagnosis of sepsis was not yet according to the new sepsis definition [31], and thus we may have missed patients because one in eight patients admitted to intensive care units with infection and new organ failure does not meet the condition of at least two systemic inflammatory response syndrome criteria.

## Conclusion

Our study showed that a PCT protocol works outside clinical studies, but these benefits along with international guideline recommendations have not yet entered the daily routine in intensive care medicine. In patients with severe sepsis and septic shock, PCT testing was irregular and adherence to a PCT protocol for discontinuation of antibiotic treatment was low, but when applied it was associated with a considerably shorter duration of antibiotic treatment and a lower antibiotic consumption for the first septic episode without negative clinical outcomes. Severity of illness, PCT peak levels, and PCT kinetics, respectively, might affect frequency of PCT testing and protocol adherence. Patients at high risk without sufficiently decreasing or even increasing PCT levels seem to prompt clinicians to continue antibiotics and to overrule PCT protocols despite reaching antibiotic stopping advice thresholds.

## Additional file

**Additional file 1: Figure S1.** Local PCT protocol. Start of antibiotic treatment is based on a clinical decision. According to this algorithm, antibiotics should be discontinued when clinical improvement goes along with decreasing PCT levels. The protocol can be overruled by the means of the attending physician due to clinical reasons or when conditions are present which require a prolonged antibiotic treatment. Daily PCT samples were recommended on day 1–3 in patients on antibiotic treatment since admission to ICU or since clinical suspicion of systemic bacterial infection. From day 4 on, PCT samples were recommended every other day in patients under antibiotic treatment.

## Abbreviations

CI: Confidence interval
ICU: Intensive care unit
pt(s): Patient(s)
PCT: Procalcitonin
SAPS: Simplified acute physiology score
SOFA: Sequential organ failure score
