# Supplementary material for: Adherence to a procalcitonin-guided antibiotic treatment protocol in patients with severe sepsis and septic shock
Source: Ann Intensive Care. 2018 Jun 4;8:68. doi: 10.1186/s13613-018-0415-5 (PMC5986690; doi:10.1186/s13613-018-0415-5)
Supplement: Supplementary file 1 — Additional file 1: Figure S1. Local PCT protocol. Start of antibiotic treatment is based on a clinical decision. According to this algorithm, antibiotics should be discontinued when clinical improvement goes along with decreasing PCT levels. The protocol can be overruled by the means of the attending physician due to clinical reasons or when conditions are present which require a prolonged antibiotic treatment. Daily PCT samples were recommended on day 1–3 in patients on antibiotic treatment since admission to ICU or since clinical suspicion of systemic bacterial infection. From day 4 on, PCT samples were recommended every other day in patients under antibiotic treatment. [file 13613_2018_415_MOESM1_ESM.docx]

**Supplemental Figure 1** Local PCT protocol. Start of antibiotic treatment is based on a clinical decision. According to this algorithm, antibiotics should be discontinued when clinical improvement goes along with decreasing PCT levels. The protocol can be overruled by the means of the attending physician due to clinical reasons or when conditions are present which require a prolonged antibiotic treatment. Daily PCT samples were recommended on day 1–3 in patients on antibiotic treatment since admission to ICU or since clinical suspicion of systemic bacterial infection. From day 4 on, PCT samples were recommended every other day in patients under antibiotic treatment.
